# Six Months vs. 12 Months of Adjuvant Trastuzumab Among Women With HER2-Positive Early-Stage Breast Cancer: A Meta-Analysis of Randomized Controlled Trials

**DOI:** 10.3389/fonc.2020.00288

**Published:** 2020-03-20

**Authors:** Huan Deng, Xianghui Du, Li Wang, Ming Chen

**Affiliations:** ^1^Institute of Cancer and Basic Medicine (ICBM), Chinese Academy of Sciences, Hangzhou, China; ^2^Department of Radiation Oncology, Cancer Hospital of the University of Chinese Academy of Sciences, Hangzhou, China; ^3^Department of Radiation Oncology, Zhejiang Key Laboratory of Radiation Oncology, Zhejiang Cancer Hospital, Hangzhou, China; ^4^College of Life Sciences, University of Chinese Academy of Sciences, Beijing, China; ^5^Key Laboratory Diagnosis and Treatment Technology on Thoracic Oncology, Zhejiang Cancer Hospital, Hangzhou, China; ^6^Jiangxi Medical College, Nanchang University, Nanchang, China

**Keywords:** optimal duration, trastuzumab, breast cancer, RCTs, meta-analysis

## Abstract

**Purpose:** Both 12 and 6 months of trastuzumab in combination with chemotherapy are effective for HER2+ early-stage breast cancer. This meta-analysis was performed to assess the effectiveness and the toxicity of the two durations.

**Methods and Materials:** We acquired relevant randomized controlled trials (RCTs) from PubMed, the Cochrane Library, ScienceDirect, EMBASE, Ovid MEDLINE, Web of Science, Scopus, and Google Scholar. Our endpoints included disease-free survival (DFS), overall survival (OS), number of recurrences, mortality and early stopping of trastuzumab, and adverse events (AEs).

**Results:** We included five good-quality studies. Both durations of trastuzumab were effective among women with HER2+ early-stage breast cancer, but 12 months of trastuzumab appeared to have better DFS [hazard ratio (HR) = 1.10, 95% confidence interval (CI): 0.99–1.23, *P* = 0.09] and better OS than 6 months of trastuzumab (HR = 1.14, 95% CI: 0.99–1.32, *P* = 0.07). However, the 12 month group had more AEs, especially cardiac events [risk ratio (RR) = 0.66, 95% CI: 0.56–0.77, *P* < 0.00001]. In our sub-analyses, the 12 months duration had better DFS among patients using trastuzumab concurrently than the 6 months duration (HR = 1.23, 95% CI: 1.06–1.44, *P* = 0.006). Additionally, the 12 months duration had superior OS in women with ER-negative breast cancer (HR = 1.51, 95% CI: 1.10–2.08, *P* = 0.01) and patients treated with trastuzumab concurrently than the 6 months duration (HR = 1.61, 95% CI: 1.13–2.29, *P* = 0.008).

**Conclusions:** Twelve months was the standard duration of adjuvant trastuzumab among patients with HER2+ early-stage breast cancer, with a tendency toward superior survival. However, patients in the 12 month group had more significant cardiac toxicity than those in the 6 month group.

## Introduction

Human epidermal growth factor receptor 2 (HER2) is overexpressed or amplified in ~15 to 20% of women with invasive breast carcinomas, which is associated with poor outcomes (1). Before the development of anti-HER2 treatments, patients with HER2+ early breast carcinomas had poor survival (2).

Trastuzumab is a type of monoclonal antibody against the HER2 receptor, and it is usually combined with other antitumor drugs for the treatment of breast carcinomas and gastric carcinomas (3). In nearly two decades, some studies have suggested that trastuzumab combined with chemotherapy may remarkably decrease the risk of relapses and/or metastasis and improve the survival of women with HER2+ breast cancer (4–8). Additionally, 1 year of trastuzumab has been commonly used as the standard duration in patients with HER2+ early-stage breast cancer (9, 10). Nevertheless, 1 year of adjuvant trastuzumab has been adopted arbitrarily, instead of based on biological evidence. Until now, the optimal duration of trastuzumab has remained controversial. To reduce the toxicity and costs for women with HER2+ breast cancer, many randomized controlled trials (RCTs) have investigated different durations of trastuzumab (11–16). However, these clinical trials reported inconsistent results for different durations vs. 12 months of trastuzumab. Two multicenter and open-label RCTs suggested that 6 months of trastuzumab failed to show non-inferiority among patients with HER2+ breast cancer compared to 12 months of trastuzumab (14, 15). However, in a prospective, phase-III RCT of 152 centers, Earl et al. indicated that 6 months of trastuzumab therapy showed non-inferiority to 1-year therapy [4-year disease-free survival (DFS): 6 month group, 89.38%; 12 month group, 89.78%], with fewer cardiac events and fewer cases of serious toxicity in the 6 month group than in the 12 month group (16).

To solve this controversy, we conducted a meta-analysis of relevant randomized trials to compare the anticancer effectiveness and toxicity of the two durations (6 and 12 months) of adjuvant trastuzumab to provide the latest evidence-based recommendations for patients with HER2+ early-stage breast cancer.

## Materials and Methods

The meta-analysis was performed in accordance with the PRISMA (Preferred Reporting Items for Systematic Review and Meta-analysis) (Table S1) guidelines (Registration information: PROSPERO CRD42019148022).

### Search Strategies

Relevant studies were found using the following databases: PubMed, ScienceDirect, Web of Science, the Cochrane Library, Scopus, EMBASE, Ovid MEDLINE, and Google Scholar. The following terms were used in the search: “6 month,” “12 month,” “trastuzumab,” and “breast cancer.” The exhaustive search strategies used in these electronic databases are displayed in Table S2. The references of all enrolled articles were searched for other eligible articles. All included articles had been published in English.

### Selection Criteria

Articles that followed the PICOS (Participants, Intervention, Control, Outcomes, Study design) selection criteria were eligible—(1) participants: women aged ≥18 years histologically diagnosed with HER2+ early-stage breast cancer in accordance with the American Society of Clinical Oncology (ASCO) guidelines (17); (2) intervention and control: 6 months vs. 12 months of adjuvant trastuzumab; (3) outcomes: DFS [defined as the date from randomization to the date of first recurrence (local or distant), the occurrence of contralateral breast cancer, the occurrence of a second non-breast primary malignancy, or death], overall survival (OS) (defined as the date from randomization to the date of death), the number of patients who relapsed, died, and stopped trastuzumab early, and adverse events (AEs); and (4) study design: RCTs published in English.

Retrospective analyses, conference abstracts, reviews without original data, meta-analyses, case reports, animal experiments, and articles with duplicate data were ineligible.

### Date Extraction

Two investigators (Deng and Du) independently extracted the following data: authors; publication date; country; number of participants; participant traits [age, menopausal status, tumor size, estrogen receptor (ER) status, nodal status, histological grade, and trastuzumab timing]; survival data (DFS, OS); number of patients who relapsed, died, and stopped trastuzumab early; and AEs. All disagreements were settled by a third investigator (Chen). When analyzing DFS and OS, we adopted the hazard ratio (HR) regarding the number and timing of the event instead of the odds ratio (OR). We obtained HRs and 95% confidence intervals (CIs) directly from studies in which univariate survival analysis was performed. Otherwise, HRs and 95% CIs were acquired from Kaplan–Meier curves (18).

### Quality Assessment

The quality of an RCT was appraised by the 5-point Jadad scale, including three main aspects: the randomization, masking, and accountability of all participants. Studies reaching 3–5 points were considered of good quality (19).

We adopted the GRADE (Grading of Recommendations Assessment, Development, and Evaluation) guidelines to assess the therapeutic strategy and study the outcomes (DFS, OS, recurrence, mortality, and toxicities). GRADE scores were categorized into four grades (high, medium, low, and very low) (20).

### Statistical Analysis

All analyses were performed using RevMan 5.2 and STATA 12.0. HRs and 95% CIs were considered when analyzing DFS and OS (an HR >1 favored the 12 months arm; an HR <1 favored the 6 months arm). The risk ratios (RRs) and 95% CIs were considered when analyzing the number of patients who relapsed, died, and stopped trastuzumab early, as well as AEs (an RR >1 favored the 12 months arm; an RR <1 favored the 6 months arm). Subanalyses of DFS and OS were conducted to determine whether the results differed according to age, menopausal status, nodal status, ER status, and trastuzumab timing. Heterogeneity was appraised using the χ^2^ test as well as the *I*^2^ statistic. When *I*^2^ was >50% or *P* was <0.10 in the χ^2^ test, showing apparent heterogeneity, we adopted a random-effect model; otherwise, we adopted a fixed-effect model. Sensitivity analyses for DFS and OS were performed to increase the robustness of the analysis. We evaluated publication bias using Begg's test and Egger's test. *P* < 0.05 was regarded as statistically significant.

## Results

### Search Results and Study Quality Appraisal

**Figure 1:**
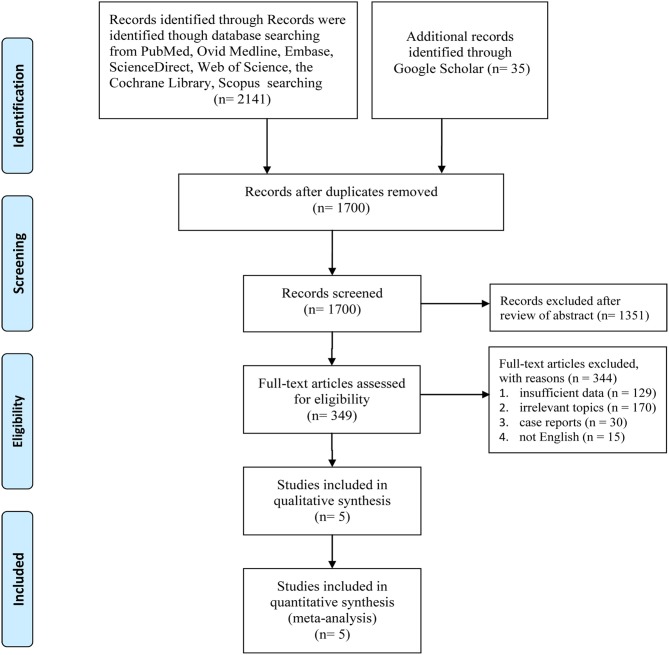
Flow chart of the study selection process.

Figure 1 displays the procedures for identifying the included studies. Ultimately, five studies involving 7,949 women with HER2+ early breast cancer (6 month group, 3,973; 12 month group, 3,976) were identified for inclusion in this meta-analysis (14–16, 21, 22). Pivot et al. published two versions of their outcomes before 2019; one reported the primary results (21), and the other reported cardiac toxicity (22), and both were derived from the PHARE trial. All included studies were of good quality (all trials scored 4 points on the Jadad scale, Table S3). Moreover, most results had high/medium GRADE scores, while some had low GRADE scores (Table S4). Table 1 shows the basic traits and the main appraisal index of all enrolled RCTs.

**Table 1:** Characteristics of all included studies.

**Study**		**Nation**	**Groups**	**Patients (*n*)**	**Median age (y)**	**MP status (*****n*****)**			**ER status**		**HER2 status**		**Herceptin timing**		**Follow-up (mo)**	**Design**	**Score^a^**
						**Pre-MP**	**Peri-MP**	**Post-MP**	**+**	**–**	**3+**	**2+**	**CC**	**SQ**			
2015	Mavroudis (14)	Greece	6 month	240	56	83	0	157	NA	NA	222	18	NA	NA	51	RCT	4
			12 month	241	54	100	0	141	NA	NA	218	13	NA	NA	47		
2019	Pivot (15)	France	6 month	1690	55	NA	NA	NA	995	695	1,546	143	961	729	7.5^b^	RCT	4
			12 month	1,690	54	NA	NA	NA	975	715	1,539	149	972	718	7.5^b^		
2019	Earl (16)	UK	6 month	2,043	56	580	150	1,070	1,411	632	1,487	497	952	1,091	5.4^b^	RCT	4
			12 month	2,045	56	567	110	1,144	1,412	6,333	1,460	540	951	1,094	5.4^b^		
2013	Pivot (21)^c^	France	6 month	1,690	55	NA	NA	NA	995	695	1,546	143	961	729	42.5	RCT	4
			12 month	1,690	54	NA	NA	NA	975	715	1,539	149	972	718	42.5		
2015	Pivot (22)^c^	France	6 month	1,690	55	NA	NA	NA	995	695	1,546	143	961	729	54.5	RCT	4
			12 month	1,690	54	NA	NA	NA	975	715	1,539	149	972	718	54.5		

*MP, menopausal; ER, estrogen receptor; HER2, human epidermal growth factor receptor; +, positive; –, negative; CC, concurrent; SQ, sequential; mo, month; NA, not available; RCT, randomized controlled trail*.

a *The quality of RCT is evaluated using the 5-point Jadad scale*.

b *The time of follow-up is calculated by year rather than month*.

c *The two studies were derived from of the PHARE trial; one reported primary results and the other one reported cardiac toxicity*.

### Survival

We evaluated the survival of patients treated with 6 and 12 months of trastuzumab in terms of DFS and OS.

**Figure 2:**
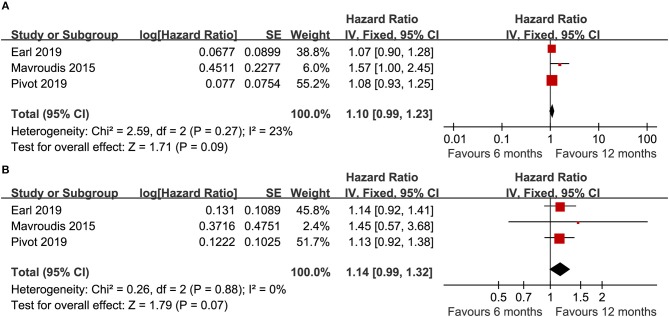
Forest plot of the DFS **(A)** and OS **(B)** associated with the 6 months group vs. the 12 months group.

Three trials (14–16) compared DFS (heterogeneity: *I*^2^ = 23%, *P* = 0.27). Although there was no significant difference, the 12 month group appeared to have improved DFS compared with the 6 month group (HR = 1.10, 95% CI: 0.99–1.23, *P* = 0.09; Figure 2A).

Three trials (14–16) compared OS (heterogeneity: *I*^2^ = 0%, *P* = 0.88). Although significant difference was not detected, the 12 month group appeared to have prolonged OS compared to the 6 month group (HR = 1.14, 95% CI: 0.99–1.32, *P* = 0.07; Figure 2B).

**Figure 3:**
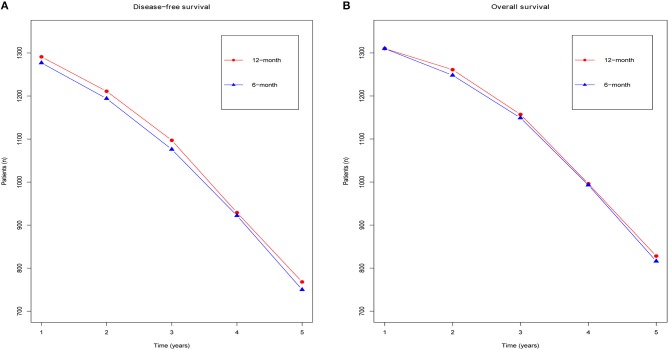
Forest plots of the yearly DFS **(A)** and yearly OS **(B)** associated with the 6 months group vs. the 12 months group.

In addition, we also made two line charts to analyze the annual DFS (Figure 3A) and OS (Figure 3B), over a time frame from 1 to 5 years (calculated from the time of randomization). Our results suggested that although obvious differences were not detected between the groups, the 12 month group appeared to have a longer survival time than the 6 month group. In particular, the DFS benefit in the 12 month group was greater than that in the 6 month group.

### Recurrence

**Figure 4:**
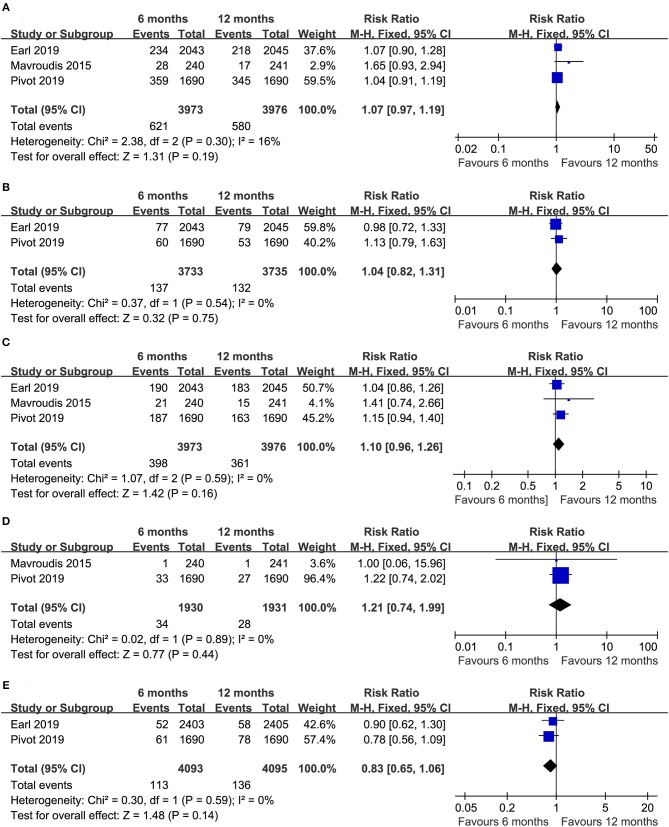
Forest plots of the RRs of total relapses **(A)**, local-regional relapse **(B)**, distant relapse **(C)**, contralateral breast cancer **(D)**, and a second primary malignancy **(E)** associated with the 6 months group vs. the 12 months group.

Three trials (14–16) compared the total relapses (heterogeneity: *I*^2^ = 16%, *P* = 0.30). Obvious differences were not detected in terms of the total relapses between the two durations of treatment (RR = 1.07, 95% CI: 0.97–1.19, *P* = 0.19; Figure 4A). In our further analysis of the total relapses, two RCTs (14–16) reported local-regional relapses, and no apparent difference was detected between the two durations of treatment (RR = 1.04, 95% CI: 0.82–1.31, *P* = 0.75; Figure 4B). Additionally, three RCTs (14–16) compared distant relapse, and there was no apparent difference (RR = 1.10, 95% CI: 0.96–1.26, *P* = 0.16; Figure 4C).

Two trials (14, 15) reported the number of women who developed contralateral breast cancer (heterogeneity: *I*^2^ = 0%, *P* = 0.89). Obvious differences were not found between the 6- and 12 month groups (RR = 1.21, 95% CI: 0.74–1.99, *P* = 0.44; Figure 4D).

Two trials (15, 16) compared the number of patients with second primary malignancies (heterogeneity: *I*^2^ = 0%, *P* = 0.59). There was no apparent difference in the development of second primary malignancies between the two durations of treatment (RR = 0.83, 95% CI: 0.65–1.06, *P* = 0.14; Figure 4E).

### Mortality

**Figure 5:**
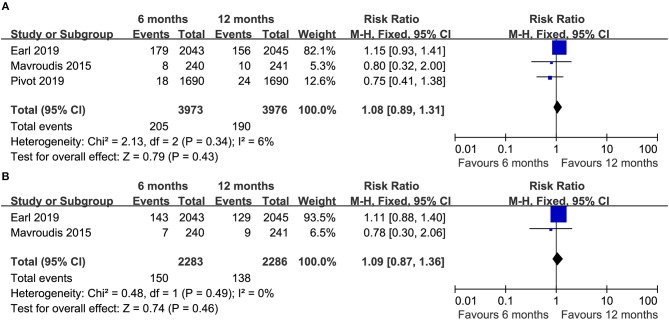
Forest plots of the total death **(A)** and death due to breast cancer **(B)** associated with the 6 months group vs. the 12 months group.

Three RCTs (14–16) reported the total number of deaths (heterogeneity: *I*^2^ = 6%, *P* = 0.34). An apparent difference was not detected between the groups (RR = 1.08, 95% CI: 0.89–1.31, *P* = 0.43; Figure 5A).

Two RCTs (14, 16) compared the number of deaths due to breast cancer, and there was no apparent difference between the two groups (RR = 1.09, 95% CI: 0.87–1.36, *P* = 0.46; Figure 5B).

### Drug Discontinuation

Some patients discontinued trastuzumab during therapy. Three RCTs (14, 16, 21) compared the number of participants who stopped trastuzumab early due to toxicity (heterogeneity: *I*^2^ = 66%, *P* = 0.05). Fewer patients in the 6 month group discontinued treatment with trastuzumab early due to toxicity than in the 12 month group (RR = 0.36, 95% CI: 0.22–0.59, *P* < 0.0001; Figure S1). Moreover, only one trial (16) reported early discontinuation of trastuzumab due to patient request, and a lower proportion of patients in the 6 month group than in the 12 month group requested the early discontinuation of trastuzumab (1 vs. 5%, *P* < 0.00001).

### Toxicity

The toxicity of adjuvant trastuzumab was compared between the 6- and 12 month groups based on grade II–IV AEs. Analyses of the five most common toxic events were conducted.

Only one study (16) reported all severe AEs, and this study demonstrated that a lower proportion of patients in the 6 month group than in the 12 month group experienced at least one severe AE (19.2 vs. 24.2%, *P* = 0.0002).

In further analysis of the five most common severe AEs (in order of incidence: cardiotoxicity, neutropenia, diarrhea, vomiting, and skin/nail toxicity), the outcomes of these AEs suggested no apparent difference in the incidence of neutropenia, diarrhea, vomiting, and skin/nail toxicity. However, three RCTs (14, 16, 22) compared cardiotoxicity (heterogeneity: *I*^2^ = 18%, *P* = 0.30), and our pooled results demonstrated that the 6 month group had a lower incidence of cardiotoxicity than the 12 month group (RR = 0.66, 95% CI: 0.56–0.77, *P* < 0.00001; Table 2).

**Table 2:** Top five severe adverse events associated with 6 month group vs. 12 month group.

**Adverse effects**	**No. of studies**	**6 month group (event/total)**	**12 month group (event/total)**	**RR (95% CI)**	***P*-value**	**Heterogeneity**	
						***I^**2**^* (%)**	***P*-value**
Cardiotoxicity	3	224/3,924	338/3,899	0.66 [0.56, 0.77]	<0.00001	18	0.30
Neutropenia	1	47/240	44/241	1.07 [0.74, 1.55]	0.71	NA	NA
Diarrhea	2	62/2,179	66/2,135	0.92 [0.65, 1.29]	0.63	9	0.30
Vomiting	2	26/2,179	28/2,135	0.92 [0.54, 1.55]	0.75	0	0.55
Skin/nail toxicity	2	23/2,179	30/2,135	0.44 [0.05, 3.68]	0.45	59	0.12

*RR, risk ratio; CI, confidence interval; NA, not available*.

### Subgroup Analysis

To determine if there were differences in survival between patients treated with 6 and 12 months of adjuvant trastuzumab, we calculated the pooled outcomes of DFS and OS according to age, menopausal status, nodal status, ER status, and trastuzumab timing (Table 3). Interestingly, the pooled results found that the 12 month group had superior DFS among breast cancer patients treated with trastuzumab concurrently (HR = 1.23, 95% CI: 1.06–1.44, *P* = 0.006). Moreover, we also found that the 12 month group had superior OS among women who had ER-negative breast cancer (HR = 1.51, 95% CI: 1.10–2.08, *P* = 0.01) and patients undergoing additional therapy concurrent with trastuzumab (HR = 1.61, 95% CI: 1.13–2.29, *P* = 0.008). Other positive results of the sub-analysis were not found.

**Table 3:** Subgroup analysis for disease-free survival and overall survival.

**Group**	**DFS**				**OS**			
	**No. of studies**	**HR (95% CI)**	**P**	***I^**2**^* (%)**	**No. of studies**	**HR (95% CI)**	***P***	***I^**2**^* (%)**
**Total**	3	1.10 [0.99, 1.23]	0.09	23	3	1.14 [0.99, 1.32]	0.07	0
**Age (y)**								
<50	3	1.09 [0.92, 1.30]	0.31	0	1	0.94 [0.64, 1.38]	0.75	NA
≥ 50	3	1.10 [0.96, 1.27]	0.17	0	1	1.25 [0.97, 1.62]	0.09	NA
**Menopausal status**								
Pre-menopausal	2	1.07 [0.79, 1.46]	0.67	0	1	0.98 [0.64, 1.51]	0.93	NA
Peri-menopausal	1	0.71 [0.31, 1.62]	0.42	NA	1	0.95 [0.32, 2.82]	0.93	NA
Post-menopausal	2	1.13 [0.90, 1.41]	0.30	12	1	1.16 [0.88, 1.53]	0.30	NA
**Nodal status**								
Positive	2	1.11 [0.92, 1.33]	0.27	0	NA	NA	NA	NA
Negative	2	1.09 [0.86, 1.39]	0.45	14	NA	NA	NA	NA
**ER status**								
Positive	3	1.04 [0.89, 1.21]	0.64	8	1	0.91 [0.68, 1.21]	0.52	NA
Negative	3	1.15 [0.99, 1.33]	0.08	0	1	1.51 [1.10, 2.08]	0.01	NA
**Trastuzumab timing**								
Concurrent	3	1.23 [1.06, 1.44]	0.006	67	1	1.61 [1.13, 2.29]	0.008	NA
Sequential	2	0.97 [0.82, 1.13]	0.66	67	1	0.93 [0.71, 1.22]	0.60	NA

*DFS, disease-free survival; OS, overall survival; y, year; ER, estrogen receptor; HR, hazard ratio; CI, confidence interval; NA, not available*.

### Sensitivity Analysis

Figure S2A clearly shows that the DFS data were robust, with no estimated value exceeding the 95% CIs. Additionally, Figure S2B demonstrates that the OS data were also robust, and no estimated value exceeded the 95% CIs.

### Publication Bias

Proof of publication bias was not detected in the DFS data (Begg's test, *P* = 1.000, Egger's test, *P* = 0.156; Figure S3A). Moreover, we could not find evidence of publication bias with Begg's test in the OS data (*P* = 0.296), while a slight publication bias was identified according to Egger's test in the OS data (*P* = 0.038, Figure S3B). This result was possibly a consequence of the limited number of enrolled RCTs, and the value might change when additional new relevant clinical trials are added.

## Discussion

Twelve months of adjuvant trastuzumab was the traditional duration for the treatment of HER2+ early-stage breast cancer (23). Six months of trastuzumab was also effective for improving the prognosis of women with HER2+ early breast cancer (24). Which duration offers more clinical benefits? Our meta-analysis compared the efficacy and toxicity of 6 vs. 12 months of trastuzumab for patients with HER2+ early-stage breast cancer. The pooled results from the included RCTs suggested that the 12 month group had a tendency toward superior survival (DFS and OS) compared to the 6 month group. No obvious differences were detected between the durations in terms of time to recurrence or death. Furthermore, 6 months was correlated with less toxicity, particularly cardiac toxicity, than 12 months of trastuzumab. In our sub-analyses, women treated with trastuzumab concurrently in the 12 month group may have had improved survival.

Survival and recurrence are the most important factors that we must take into account in regard to the therapeutic efficacy of both durations of trastuzumab. The pooled outcomes indicated that obvious differences were not detected in terms of relapse and death, but the HR was more reliable than the RR, because the HR simultaneously considered the numbers and time of events. Furthermore, our results showed that although obvious differences were not detected, patients in the 12 month group appeared to have superior DFS and OS. In a recent retrospective analysis, Arif et al. reported that participants in the 1-year group had a longer median DFS than those in the 6 month group (63 vs. 54 months, *P* = 0.048), and the 1-year group had a tendency toward a superior median OS (62 vs. 57 months, *P* = 0.073) (25). Similarly, some phase III clinical trials have compared 9 weeks and the standard duration (1 year) of trastuzumab, and these RCTs demonstrated that a short duration (9 weeks) of trastuzumab was not non-inferior to 12 months of trastuzumab in terms of the anticancer effectiveness (11, 12). In addition, a similar meta-analysis also showed that 1 year of trastuzumab had superior OS (HR = 1.22, 95% CI: 1.07–1.39, *P* = 0.003) and better DFS (HR = 1.19, 95% CI: 1.08–1.30, *P* < 0.001) compared to short durations (6 months and 9 weeks) among patients with HER2+ early breast cancer (26). Two included trials (14, 15) suggested similar outcomes, apart from the PERSEPHONE trial (16), but they could not confirm the non-inferiority of 6 months of trastuzumab. Notably, we found several differences in our included RCTs, which might explain the non-inferiority demonstrated in the PERSEPHONE trial. First, ~72% of participants had 3+ positivity for HER2 in the PERSEPHONE trial, which was relatively lower than the nearly 91% of participants who had this feature in the HORG and PHARE trials. Second, nearly 69% of breast cancer patients were ER+ in the PERSEPHONE trial, and this proportion was relatively higher than those in the HORG and PHARE trials (58 to 65%). Third, the number of patients with node-negative breast cancer was higher in the PERSEPHONE trial (60%) than in the HORG trial (21%) and PHARE trial (54%). Therefore, we assumed that the non-inferiority identified in the PERSEPHONE trial may be a result of the inclusion of more low-risk participants. Consequently, 12 months of trastuzumab might be more suitable for women with high-risk breast cancer, and 6 months might be more suitable for low-risk breast cancer patients. These conclusions require cautious acceptance and further confirmation.

Clearly, the subgroup analysis also played a key role in the evaluation of the therapeutic effect between both durations. Our sub-analysis suggested that the 12 month duration resulted in improved DFS in patients treated with trastuzumab concurrently, and the 12 month duration had superior OS among participants treated with trastuzumab in addition to concurrent therapy and participants with ER-negative breast cancer. A multicenter phase III RCT also showed that the 1-year duration of treatment was associated with an improved DFS (HR = 1.53, 95% CI: 1.16–2.01, *P* = 0.001) and better OS (HR = 1.61, 95% CI: 1.13–2.29, *P* = 0.008) among early breast cancer participants treated with trastuzumab concurrently (16). Furthermore, a recent meta-analysis with six RCTs compared the therapeutic effect and toxicity between short durations (9 weeks and 6 months) and 1 year of trastuzumab, and the sub-analyses also showed that patients treated with trastuzumab concurrently in the 1-year group had an improved DFS (HR = 1.22, 95% CI: 1.09–1.38, *P* = 0.0008) (27). In conclusion, improved survival was more evident among patients treated with concurrent trastuzumab and with ER-negative breast cancer.

Toxicity is an essential factor when comparing safety between the 6 month and 1-year durations among participants with HER2+ early-stage breast cancer. Our results showed that participants in the 6 month group had a lower incidence rate of AEs, particularly cardiac toxicity, than those in the 12 month group. In addition, we also found that the 6 month group had a lower proportion of patients with early discontinuation of trastuzumab due to AEs than the 12 month group, which suggested reduced toxicity in the 6 month group. Undoubtedly, cardiac toxicity was the most significant and serious toxic event for breast cancer patients treated with trastuzumab (28–30). Meanwhile, the pooled outcomes demonstrated no obvious differences in terms of other AEs. Therefore, we focused on the cardiac toxicity of patients using trastuzumab. In fact, cardiac toxicity is defined as two conditions—(1) low left ventricular ejection fraction (LVEF): an absolute reduction in LVEF by <50% regardless of the baseline LVEF or an absolute reduction of 10% from baseline when LVEF at baseline was no more than 50%; and (2) clinical cardiac dysfunction: symptoms or signs of congestive heart failure or use of new medication for heart disease (31–33). In a recent study on cardiotoxicity, Pivot et al. suggested that the occurrence of cardiac dysfunction in the 6 month group was lower than that in the 1-year group (3.43% vs. 5.92%, *P* = 0.001) (22). After 8 years of follow-up, a randomized trial demonstrated an obvious LVEF reduction in the 1-year group that was apparently lower than that in the 2-year group (4.10% vs. 7.17%, *P* < 0.001), and fewer patients in the 1-year group discontinued trastuzumab due to cardiotoxicity than in the 2-year group (5.23 vs. 9.38%) (34). Therefore, we assume that cardiotoxicity may be more frequent and serious if the duration of trastuzumab is longer. Briefly, a short duration of adjuvant trastuzumab would be more suitable for breast cancer patients with previous heart disease or patients who previously experienced cardiotoxicity during chemotherapy.

The medical costs incurred by breast cancer patients are also an important consideration. Obviously, due to its shorter duration, 6 months of trastuzumab would cost less money compared with 12 months. In addition, a recent study from Iran indicated that 6 months of trastuzumab was a more cost-effective choice than 12 months, with far less incremental spending (8,826 vs. 18,588 pounds) (35). As a result, 6 months of trastuzumab might decrease the financial pressure on thousands of families using trastuzumab, and reduce their psychological burdens in the face of huge medical costs; this shorter duration of trastuzumab is more likely to be affordable for patients in low- and middle-income countries who lack sufficient medical funds.

Admittedly, our meta-analysis is not the first to compare short durations vs. 12 months of trastuzumab, and there are at least six similar meta-analyses or systematic reviews so far (26, 27, 36–39). However, these similar studies were found to have some serious limitations. Firstly, the two studies presented at ASCO 2019 provided only brief abstracts rather than detailed full-text articles, and they may lack some important information (36, 37). Secondly, most similar studies compared short durations vs. 12 months of trastuzumab, but we concentrated on 6 months of trastuzumab rather than several different short durations (26, 27, 36–39). There may be substantial bias if 6 months and other different short durations are combined arbitrarily. Thirdly, brief abstracts of some relevant RCTs were included in similar meta-analyses published before June 2019 (26, 27, 36–39), because the latest results of two key RCTs (15, 16) were published in the *Lancet* journal in June 2019; this may have substantially decreased the reliability of their final outcomes. Fourthly, previous meta-analyses (36–39) lacked relevant registration information in PROSPERO, which was indispensable for performing meta-analyses. However, our meta-analysis has some irreplaceable advantages compared with previous similar meta-analyses: (1) The detailed full text was provided in our meta-analysis, and ours could supplement some important information compared with the two previous meta-analyses (36, 37). (2) We only concentrated on 6 months of trastuzumab rather than several different short durations, so our meta-analysis had greater accuracy and possibly less bias than most similar meta-analyses (26, 27, 36–39). (3) Unlike most similar meta-analyses (26, 27, 36–39), we included the full texts of all relevant RCTs, and we made full use of the full texts and Supplementary Materials of these RCTs. (4) Compared with other studies (36–39), our meta-analysis was the first one that provided relevant registration information in PROSPERO.

There were several limitations of this study. First, although all enrolled articles were RCTs, the absolute number of enrolled clinical trials (only three RCTs) might influence the quality of the results. Second, some outcomes, particularly skin/nail toxicity and early discontinuation of trastuzumab due to AEs, had apparent heterogeneity (59 and 66%, respectively), which may have affected the quality of the results. Third, the number of participants in the two durations of trastuzumab was relatively small, which have caused relatively unreliable estimated values. Fourth, some studies had low GRADE scores, which may have weakened the quality of these results. Fifth, we could not perfectly match samples for various confounding factors (such as histological types), and this may have had an impact on the pooled outcomes.

## Conclusion

Twelve months of adjuvant trastuzumab seems to be more appropriate in women with HER2+ early-stage breast cancer than 6 months of adjuvant trastuzumab, with a trend toward superior DFS and OS, especially for women using trastuzumab concurrently and women with ER-negative breast cancer. However, it is noteworthy that 12 months of trastuzumab has more frequent and severe cardiac toxicities than 6 months of trastuzumab. Additionally, 6 months of trastuzumab may be more suitable for patients who have a history of heart disease, patients who previously experienced cardiotoxicity during chemotherapy, and low-income patients who have to consider medical costs.

## Data Availability Statement

All datasets generated for this study are included in the article/Supplementary Material.

## Author Contributions

HD had full access to all of the data in the manuscript and takes responsibility for the integrity of the data and the accuracy of the data analysis. HD and MC: drafting of the manuscript. HD and LW: statistical analysis. XD and MC: supervision. All authors: concept and design, acquisition, analysis, interpretation of data, and critical revision of the manuscript for important intellectual content.

### Conflict of Interest

The authors declare that the research was conducted in the absence of any commercial or financial relationships that could be construed as a potential conflict of interest.

## Supplementary Material

Figure S1 Forest plots of early discontinuation of trastuzumab due to toxicity associated with the 6 month group vs. the 12 month group.

Figure S2 Sensitivity analysis of DFS **(A)** and OS **(B)**.

Figure S3 Begg's and Egger's tests for comparisons of DFS **(A)** and OS **(B)** between the 6 month group and the 12 month group.

Table S1 PRISMA 2009 Checklist.

Table S2 Search strategy.

Table S3 Quality assessment of all included studies.

Table S4 GRADE quality assessment according to therapeutic strategy and study design for DFS, OS, recurrence, mortality, and toxicity.
