# Supplementary figures and images for: Six Months vs. 12 Months of Adjuvant Trastuzumab Among Women With HER2-Positive Early-Stage Breast Cancer: A Meta-Analysis of Randomized Controlled Trials
Source: Front Oncol. 2020 Mar 20;10:288. doi: 10.3389/fonc.2020.00288 (PMC7098966; doi:10.3389/fonc.2020.00288)

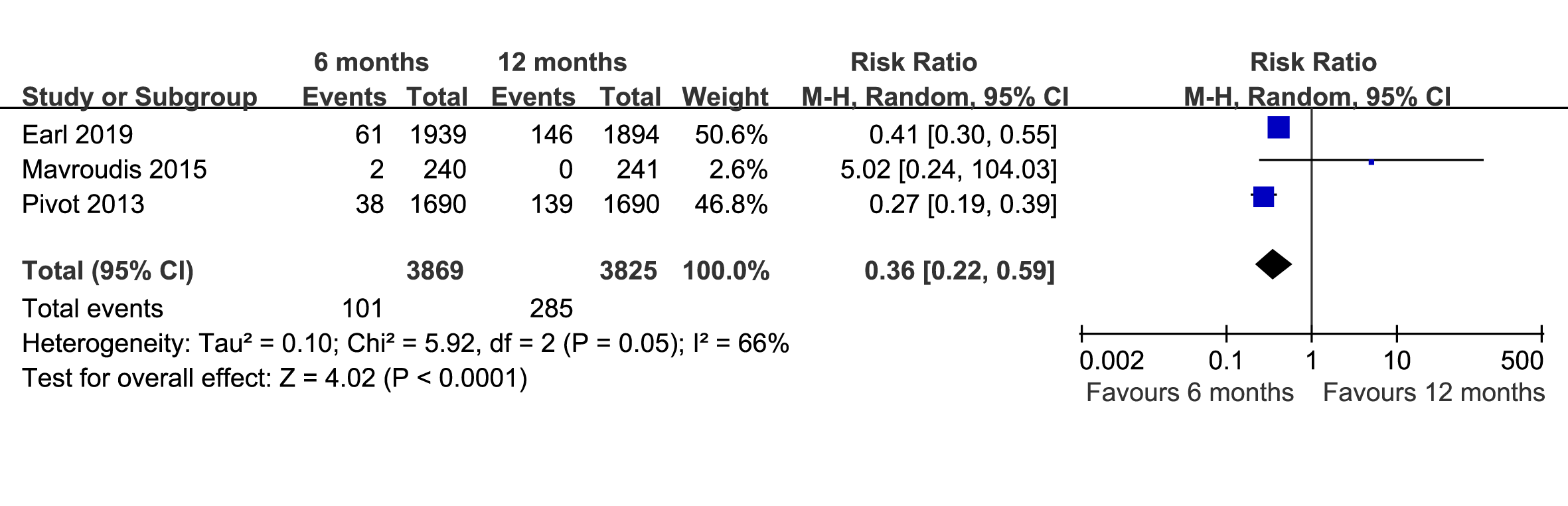

Supplement: Figure S1 — Forest plots of early discontinuation of trastuzumab due to toxicity associated with the 6 month group vs. the 12 month group. [file Image_1.TIF]

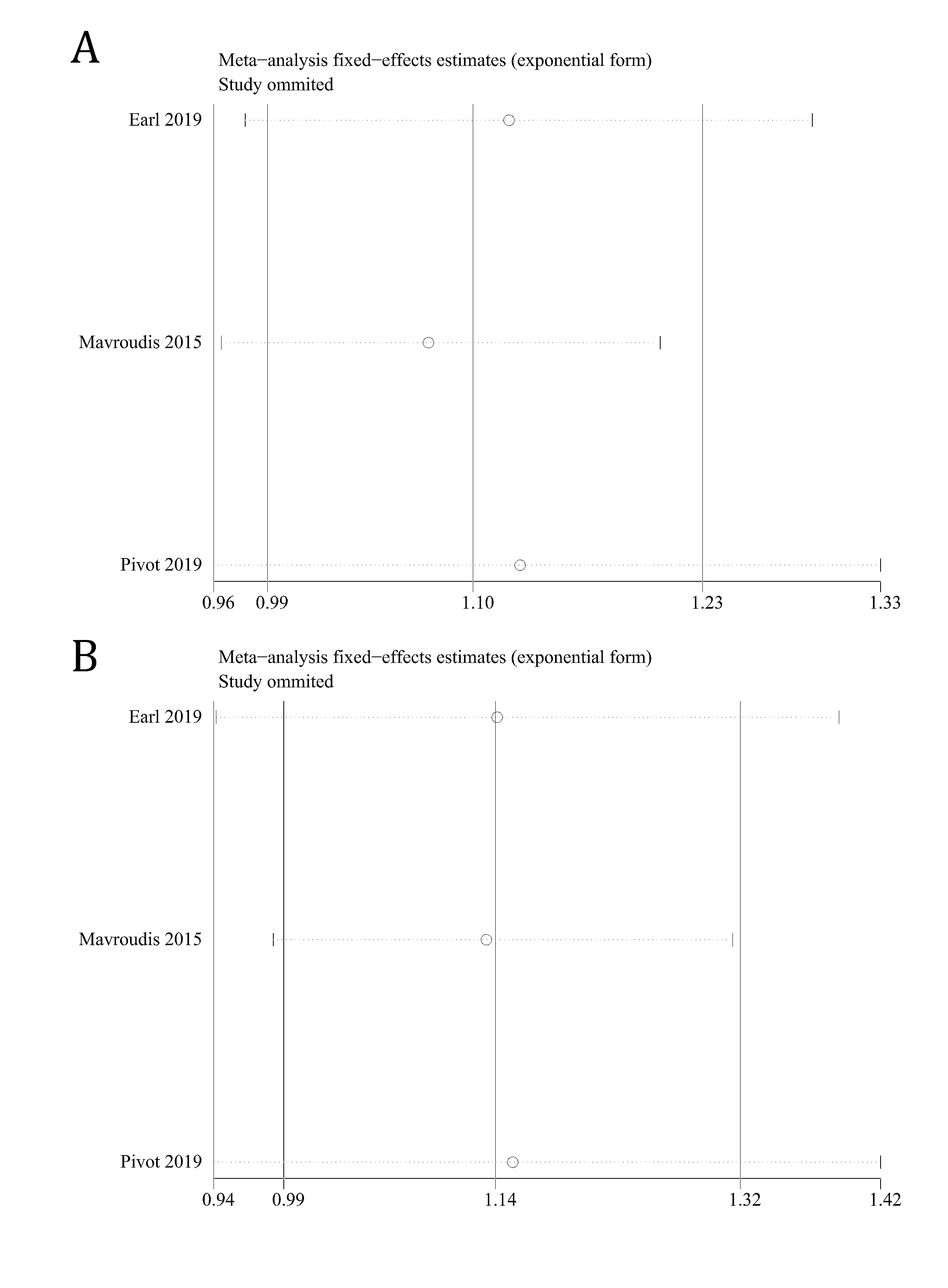

Supplement: Figure S2 — Sensitivity analysis of DFS (A) and OS (B). [file Image_2.TIF]

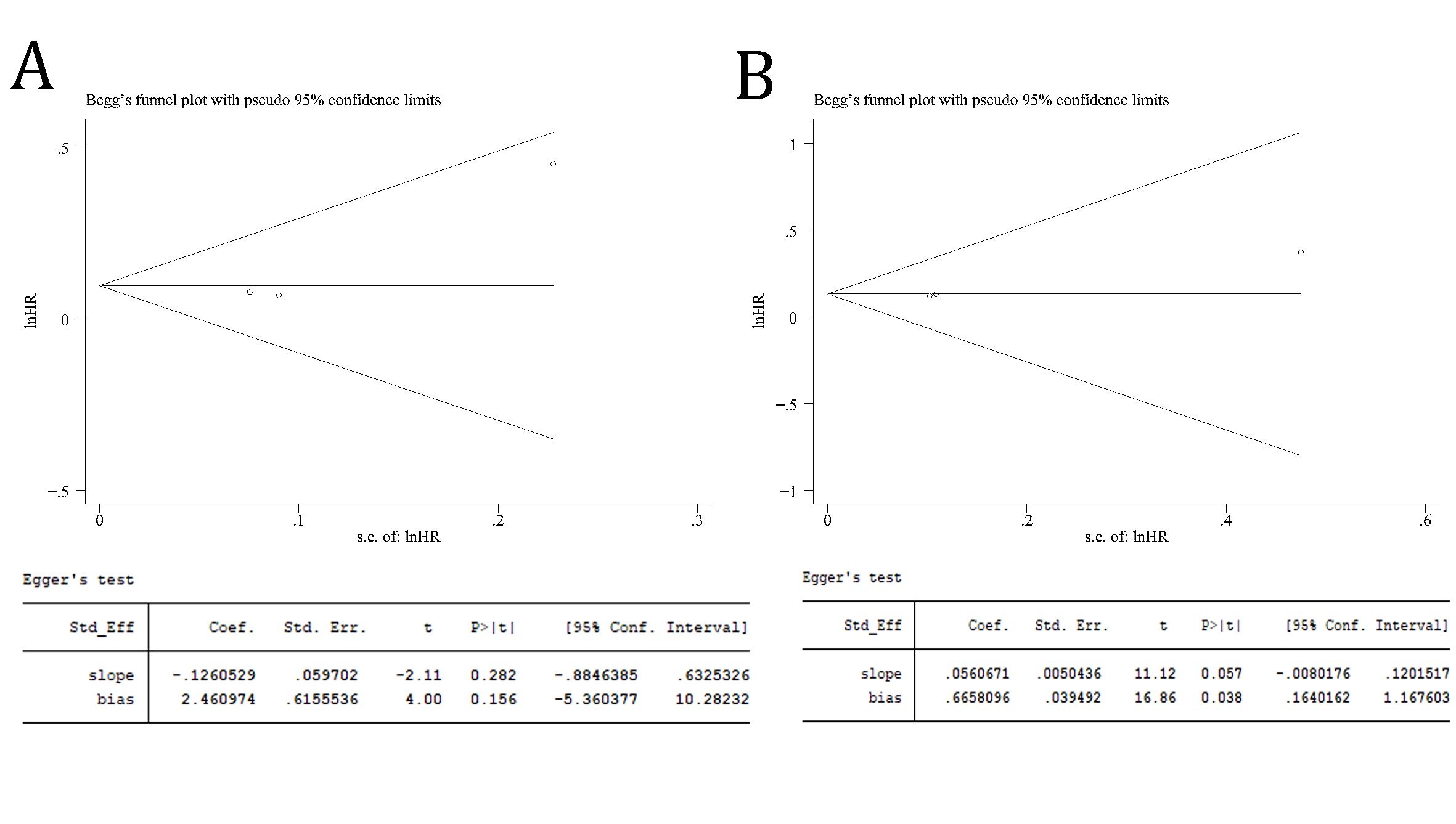

Supplement: Figure S3 — Begg's and Egger's tests for comparisons of DFS (A) and OS (B) between the 6 month group and the 12 month group. [file Image_3.TIF]
