# Supplementary material for: Six Months vs. 12 Months of Adjuvant Trastuzumab Among Women With HER2-Positive Early-Stage Breast Cancer: A Meta-Analysis of Randomized Controlled Trials
Source: Front Oncol. 2020 Mar 20;10:288. doi: 10.3389/fonc.2020.00288 (PMC7098966; doi:10.3389/fonc.2020.00288)
Supplement: Table S2 — Search strategy. [file Table_2.DOCX]

**Appendix 2. Search strategy**

The combined text and medical subject heading (MeSH) terms used were: “Breast Neoplasms”, “Trastuzumab”, “6 months”, and “12 months”.

**PubMed**

The database was searched on August 6, 2019, n= 376.

Search Strategy:

(Breast Neoplasms [MeSH term] OR Breast Neoplasms [Text Word] OR Breast Neoplasm [Text Word] OR Neoplasm, Breast [Text Word] OR Breast Tumors [Text Word] OR Breast Tumor [Text Word] OR Tumor, Breast [Text Word] OR Tumors, Breast [Text Word] OR Neoplasms, Breast [Text Word] OR Breast Cancer [Text Word] OR Cancer, Breast [Text Word] OR Mammary Cancer [Text Word] OR Cancer, Mammary [Text Word] OR Cancers, Mammary [Text Word] OR Mammary Cancers [Text Word] OR Malignant Neoplasm of Breast [Text Word] OR Breast Malignant Neoplasm [Text Word] OR Breast Malignant Neoplasms [Text Word] OR Malignant Tumor of Breast [Text Word] OR Breast Malignant Tumor [Text Word] OR Breast Malignant Tumors [Text Word] OR Cancer of Breast [Text Word] OR Cancer of the Breast [Text Word] OR Mammary Carcinoma, Human [Text Word] OR Carcinoma, Human Mammary [Text Word] OR Carcinomas, Human Mammary [Text Word] OR Human Mammary Carcinomas [Text Word] OR Mammary Carcinomas, Human [Text Word] OR Human Mammary Carcinoma [Text Word] OR Mammary Neoplasms, Human [Text Word] OR Human Mammary Neoplasm [Text Word] OR Human Mammary Neoplasms [Text Word] OR Neoplasm, Human Mammary [Text Word] OR Neoplasms, Human Mammary [Text Word] OR Mammary Neoplasm, Human [Text Word] OR Breast Carcinoma [Text Word] OR Breast Carcinomas [Text Word] OR Carcinoma, Breast [Text Word] OR Carcinomas, Breast [Text Word]) AND (Trastuzumab [MeSH term] OR Trastuzumab [Text Word] OR Herceptin [Text Word]) AND (6 months [Text Word] OR six months [Text Word]) AND (12 months [Text Word] OR 1 year [Text Word]).

**Web of Science**

The database was searched on August 6, 2019, n= 72.

Search Strategy:

1 TOPIC: (“Breast Neoplasms” OR “Breast Neoplasm” OR “Neoplasm, Breast” OR “Breast Tumors” OR “Breast Tumor” OR “Tumor, Breast” OR “Tumors, Breast” OR “Neoplasms, Breast” OR “Breast Cancer” OR “Cancer, Breast” OR “Mammary Cancer” OR “Cancer, Mammary” OR “Cancers, Mammary” OR “Mammary Cancers” OR “Malignant Neoplasm of Breast” OR “Breast Malignant Neoplasm” OR “Breast Malignant Neoplasms” OR “Malignant Tumor of Breast” OR “Breast Malignant Tumor” OR “Breast Malignant Tumors” OR “Cancer of Breast” OR “Cancer of the Breast” OR “Mammary Carcinoma, Human” OR “Carcinoma, Human Mammary” OR “Carcinomas, Human Mammary” OR “Human Mammary Carcinomas” OR “Mammary Carcinomas, Human” OR “Human Mammary Carcinoma” OR “Mammary Neoplasms, Human” OR “Human Mammary Neoplasm” OR “Human Mammary Neoplasms” OR “Neoplasm, Human Mammary” OR “Neoplasms, Human Mammary” OR “Mammary Neoplasm, Human” OR “Breast Carcinoma” OR “Breast Carcinomas” OR “Carcinoma, Breast” OR “Carcinomas, Breast”)

2 TOPIC: (“Trastuzumab” OR “Herceptin”)

3 TOPIC: (“6 months” OR “six months”)

4 TOPIC: (“12 months” OR “1 year”)

5 #1 AND #2 AND #3 AND #4 (n= 72)

**EMBASE**

The database was searched on August 6, 2019, n= 227.

Search Strategy:

(‘Breast Neoplasms’:ti,ab,kw OR ‘Breast Neoplasm’:ti,ab,kw OR ‘Neoplasm, Breast’:ti,ab,kw OR ‘Breast Tumors’:ti,ab,kw OR ‘Breast Tumor’:ti,ab,kw OR ‘Tumor, Breast’:ti,ab,kw OR ‘Tumors, Breast’:ti,ab,kw OR ‘Neoplasms, Breast’:ti,ab,kw OR ‘Breast Cancer’:ti,ab,kw OR ‘Cancer, Breast’:ti,ab,kw OR ‘Mammary Cancer’:ti,ab,kw OR ‘Cancer, Mammary’:ti,ab,kw OR ‘Cancers, Mammary’:ti,ab,kw OR ‘Mammary Cancers’:ti,ab,kw OR ‘Malignant Neoplasm of Breast’:ti,ab,kw OR ‘Breast Malignant Neoplasm’:ti,ab,kw OR ‘Breast Malignant Neoplasms’:ti,ab,kw OR ‘Malignant Tumor of Breast’:ti,ab,kw OR ‘Breast Malignant Tumor’:ti,ab,kw OR ‘Breast Malignant Tumors’:ti,ab,kw OR ‘Cancer of Breast’:ti,ab,kw OR ‘Cancer of the Breast’:ti,ab,kw OR ‘Mammary Carcinoma, Human’:ti,ab,kw OR ‘Carcinoma, Human Mammary’:ti,ab,kw OR ‘Carcinomas, Human Mammary’:ti,ab,kw OR ‘Human Mammary Carcinomas’:ti,ab,kw OR ‘Mammary Carcinomas, Human’:ti,ab,kw OR ‘Human Mammary Carcinoma’:ti,ab,kw OR ‘Mammary Neoplasms, Human’:ti,ab,kw OR ‘Human Mammary Neoplasm’:ti,ab,kw OR ‘Human Mammary Neoplasms’:ti,ab,kw OR ‘Neoplasm, Human Mammary’:ti,ab,kw OR ‘Neoplasms, Human Mammary’:ti,ab,kw OR ‘Mammary Neoplasm, Human’:ti,ab,kw OR ‘Breast Carcinoma’:ti,ab,kw OR ‘Breast Carcinomas’:ti,ab,kw OR ‘Carcinoma, Breast’:ti,ab,kw OR ‘Carcinomas, Breast’:ti,ab,kw) AND (‘Trastuzumab’:ti,ab,kw OR ‘Herceptin’:ti,ab,kw) AND (‘6 months’:ti,ab,kw OR ‘six months’:ti,ab,kw) AND (‘12 months’:ti,ab,kw OR ‘1 year’:ti,ab,kw)

**Cochrane Library**

The database was searched on August 6, 2019, n= 393.

Search Strategy:

(“Breast Neoplasms” OR “Breast Neoplasm” OR “Neoplasm, Breast” OR “Breast Tumors” OR “Breast Tumor” OR “Tumor, Breast” OR “Tumors, Breast” OR “Neoplasms, Breast” OR “Breast Cancer” OR “Cancer, Breast” OR “Mammary Cancer” OR “Cancer, Mammary” OR “Cancers, Mammary” OR “Mammary Cancers” OR “Malignant Neoplasm of Breast” OR “Breast Malignant Neoplasm” OR “Breast Malignant Neoplasms” OR “Malignant Tumor of Breast” OR “Breast Malignant Tumor” OR “Breast Malignant Tumors” OR “Cancer of Breast” OR “Cancer of the Breast” OR “Mammary Carcinoma, Human” OR “Carcinoma, Human Mammary” OR “Carcinomas, Human Mammary” OR “Human Mammary Carcinomas” OR “Mammary Carcinomas, Human” OR “Human Mammary Carcinoma” OR “Mammary Neoplasms, Human” OR “Human Mammary Neoplasm” OR “Human Mammary Neoplasms” OR “Neoplasm, Human Mammary” OR “Neoplasms, Human Mammary” OR “Mammary Neoplasm, Human” OR “Breast Carcinoma” OR “Breast Carcinomas” OR “Carcinoma, Breast” OR “Carcinomas, Breast”):ti,ab,kw AND ("sunitinib" OR "sunitinib malate" OR "Sutent" OR "SU 11248" OR "SU 011248"):ti,ab,kw AND (“Trastuzumab” OR “Herceptin”): ti,ab,kw AND (“6 months” OR “six months”):ti,ab,kw AND (“12 months” OR “1 year”):ti,ab,kw- (Word variations have been searched)

**Ovid MEDLINE**

The database was searched on August 6, 2019, n= 52.

Search Strategy:

1 Breast Neoplasms

2 Breast Neoplasm

3 Neoplasm, Breast

4 Breast Tumors

5 Breast Tumor

6 Tumor, Breast

7 Tumors, Breast

8 Neoplasms, Breast

9 Breast Cancer

10 Cancer, Breast

11 Mammary Cancer

12 Cancer, Mammary

13 Cancers, Mammary

14 Mammary Cancers

15 Malignant Neoplasm of Breast

16 Breast Malignant Neoplasm

17 Breast Malignant Neoplasms

18 Malignant Tumor of Breast

19 Breast Malignant Tumor

20 Breast Malignant Tumors

21 Cancer of Breast

22 Cancer of the Breast

23 Mammary Carcinoma, Human

24 Carcinoma, Human Mammary

25 Carcinomas, Human Mammary

26 Human Mammary Carcinomas

27 Mammary Carcinomas, Human

28 Human Mammary Carcinoma

29 Mammary Neoplasms, Human

30 Human Mammary Neoplasm

31 Human Mammary Neoplasms

32 Neoplasm, Human Mammary

33 Neoplasms, Human Mammary

34 Mammary Neoplasm, Human

35 Breast Carcinoma

36 Breast Carcinomas

37 Carcinoma, Breast

38 Carcinomas, Breast

39 or/1-18 [Breast Neoplasms]

40 Trastuzumab

41Herceptin

42 or/40-41[Trastuzumab]

43 6 months

44 six months

45 or/43-44 [6 months]

46 12 months

47 1 year

48 or/46-47[12 months]

49 39 and 42 and 45 and 48

30 limit 49 to humans (51)

**ScienceDirect**

The database was searched on August 6, 2019, n= 1839. 只导出622篇

Search Strategy:

Title, abstract, keywords: ((“Breast Neoplasms” OR “Breast Neoplasm” OR “Neoplasm, Breast” OR “Breast Tumors” OR “Breast Tumor” OR “Tumor, Breast” OR “Tumors, Breast” OR “Neoplasms, Breast” OR “Breast Cancer” OR “Cancer, Breast” OR “Mammary Cancer” OR “Cancer, Mammary” OR “Cancers, Mammary” OR “Mammary Cancers” OR “Malignant Neoplasm of Breast” OR “Breast Malignant Neoplasm” OR “Breast Malignant Neoplasms” OR “Malignant Tumor of Breast” OR “Breast Malignant Tumor” OR “Breast Malignant Tumors” OR “Cancer of Breast” OR “Cancer of the Breast” OR “Mammary Carcinoma, Human” OR “Carcinoma, Human Mammary” OR “Carcinomas, Human Mammary” OR “Human Mammary Carcinomas” OR “Mammary Carcinomas, Human” OR “Human Mammary Carcinoma” OR “Mammary Neoplasms, Human” OR “Human Mammary Neoplasm” OR “Human Mammary Neoplasms” OR “Neoplasm, Human Mammary” OR “Neoplasms, Human Mammary” OR “Mammary Neoplasm, Human” OR “Breast Carcinoma” OR “Breast Carcinomas” OR “Carcinoma, Breast” OR “Carcinomas, Breast”) AND (“Trastuzumab” OR “Herceptin”) AND (“6 months” OR “six months”) AND (“12 months” OR “1 year”))

**Scopus**

The database was searched on August 6, 2019, n= 434.

Search Strategy:

TITLE-ABS-KEY ((“Breast Neoplasms” OR “Breast Neoplasm” OR “Neoplasm, Breast” OR “Breast Tumors” OR “Breast Tumor” OR “Tumor, Breast” OR “Tumors, Breast” OR “Neoplasms, Breast” OR “Breast Cancer” OR “Cancer, Breast” OR “Mammary Cancer” OR “Cancer, Mammary” OR “Cancers, Mammary” OR “Mammary Cancers” OR “Malignant Neoplasm of Breast” OR “Breast Malignant Neoplasm” OR “Breast Malignant Neoplasms” OR “Malignant Tumor of Breast” OR “Breast Malignant Tumor” OR “Breast Malignant Tumors” OR “Cancer of Breast” OR “Cancer of the Breast” OR “Mammary Carcinoma, Human” OR “Carcinoma, Human Mammary” OR “Carcinomas, Human Mammary” OR “Human Mammary Carcinomas” OR “Mammary Carcinomas, Human” OR “Human Mammary Carcinoma” OR “Mammary Neoplasms, Human” OR “Human Mammary Neoplasm” OR “Human Mammary Neoplasms” OR “Neoplasm, Human Mammary” OR “Neoplasms, Human Mammary” OR “Mammary Neoplasm, Human” OR “Breast Carcinoma” OR “Breast Carcinomas” OR “Carcinoma, Breast” OR “Carcinomas, Breast”) AND (“Trastuzumab” OR “Herceptin”) AND (“6 months” OR “six months”) AND (“12 months” OR “1 year”).
