# Supplementary material for: Six Months vs. 12 Months of Adjuvant Trastuzumab Among Women With HER2-Positive Early-Stage Breast Cancer: A Meta-Analysis of Randomized Controlled Trials
Source: Front Oncol. 2020 Mar 20;10:288. doi: 10.3389/fonc.2020.00288 (PMC7098966; doi:10.3389/fonc.2020.00288)
Supplement: Table S3 — Quality assessment of all included studies. [file Table_3.DOCX]

**Table S3** Quality assessment of all included studies.

| **RCTs** | | **Randomization** | **Masking** | **Accountability of all patients** | **Quality (score) ^a^** |
| --- | --- | --- | --- | --- | --- |
|  |  |  |  |  |  |
| 2015 | Mavroudis [13] | ★★ | ★ | ★ | 4 |
| 2019 | Earl [14] | ★★ | ★ | ★ | 4 |
| 2019 | Pivot [15] | ★★ | ★ | ★ | 4 |
| 2013 | Pivot [20] **^b^** | ★★ | ★ | ★ | 4 |
| 2015 | Pivot [21] **^b^** | ★★ | ★ | ★ | 4 |

**Abbreviations:** RCTs: randomized controlled trials.

a The quality of RCTs were evaluated using the 5-point Jadad scale.

b The two studies were the early versions of the PHARE trial, one reported primary results and the other one reported cardiac toxicity.
