# Supplementary material for: Six Months vs. 12 Months of Adjuvant Trastuzumab Among Women With HER2-Positive Early-Stage Breast Cancer: A Meta-Analysis of Randomized Controlled Trials
Source: Front Oncol. 2020 Mar 20;10:288. doi: 10.3389/fonc.2020.00288 (PMC7098966; doi:10.3389/fonc.2020.00288)
Supplement: Table S4 — GRADE quality assessment according to therapeutic strategy and study design for DFS, OS, recurrence, mortality, and toxicity. [file Table_4.DOCX]

**Table S4** GRADE Quality assessment by therapeutic strategy and study design for the outcomes (DFS, OS, recurrence, mortality, and toxicity).

| **Primary outcomes** | **No. of Studies** | | **No. of participants** | | **Differences ^a^（95%CI）** | **Quality assessment** | | | | | | **Quality** |
| --- | --- | --- | --- | --- | --- | --- | --- | --- | --- | --- | --- | --- |
|  |  |  | **6-month group** | **12-month group** |  | **Risk of bias^b^** | **Inconsistency** | **Indirectness** | **Imprecision** | **Publication bias ^c^** | |  |
| **Survival** |  | |  |  |  |  |  |  |  |  | |  |
| DFS | 3 | | 3973/3973 | 3976/3976 | 1.10 [0.99, 1.23] | No | Serious (-1) | No indirectness | No imprecision | | Unlikely | medium |
| OS | 3 | | 3973/3973 | 3976/3976 | 1.14 [0.99, 1.32] | No | No inconsistency | No indirectness | No imprecision | | Unlikely | high |
| **Recurrence** |  | |  |  |  |  |  |  |  | |  |  |
| Total relapses | 3 | | 621/3973 | 580/3976 | 1.07 [0.97, 1.19] | No | No inconsistency | No indirectness | No imprecision | | Serious (-1) | medium |
| Local-regional relapse | 2 | | 137/3733 | 132/3735 | 1.04 [0.82, 1.31] | No | No inconsistency | No indirectness | No imprecision | | Unlikely | high |
| Distant relapse | 3 | | 398/3973 | 361/3976 | 1.10 [0.96, 1.26] | No | No inconsistency | No indirectness | No imprecision | | Unlikely | high |
| Contralateral breast cancer | 2 | | 34/1930 | 28/1931 | 1.21 [0.74, 1.99] | No | No inconsistency | No indirectness | No imprecision | | Serious (-1) | medium |
| Second primary malignancy | | 2 | 113/4093 | 136/4095 | 0.83 [0.65, 1.06] | No | No inconsistency | No indirectness | No imprecision | | Unlikely | high |
| **Mortality** |  | |  |  |  |  |  |  |  | |  |  |
| Total death | 3 | | 205/3973 | 190/3976 | 1.08 [0.89, 1.31] | No | Serious (-1) | No indirectness | No imprecision | | Serious (-1) | low |
| Death due to breast cancer | 2 | | 150/2283 | 138/2286 | 1.09 [0.87, 1.36] | No | No inconsistency | No indirectness | No imprecision | | Unlikely | high |
| **Drug discontinuance** |  | |  |  |  |  |  |  |  | |  |  |
| Early stopping of trastuzumab due to AEs | 3 | | 101/3689 | 285/3825 | 0.36 [0.22, 0.59] | No | Serious (-1) | No indirectness | No imprecision | | Serious (-1) | low |
| **Toxicity** |  | |  |  |  |  |  |  |  | |  |  |
| Cardiotoxicity | 3 | | 224/3924 | 338/3899 | 0.66 [0.56, 0.77] | No | Serious (-1) | No indirectness | No imprecision | | Serious (-1) | low |
| Neutropenia | 1 | | 47/240 | 44/241 | 1.07 [0.74, 1.55] | No | No inconsistency | No indirectness | No imprecision | | Unlikely | high |
| Diarrhea | 2 | | 62/2179 | 66/2135 | 0.92 [0.65, 1.29] | No | No inconsistency | No indirectness | No imprecision | | Serious (-1) | medium |
| Vomiting | 2 | | 26/2179 | 28/2135 | 0.92 [0.54, 1.55] | No | No inconsistency | No indirectness | No imprecision | | Unlikely | high |
| Skin/nail toxicity | 2 | | 23/2179 | 30/2135 | 0.44 [0.05, 3.68] | No | Serious (-1) | No indirectness | No imprecision | | Unlikely | medium |

**Abbreviations**: DFS: disease-free survival; OS: overall survival; HR: hazard ratio; RR: risk ratio; CI: confidence interval; AEs: adverse events.

a Differences: hazard ratio(HR) for DFS and OS; risk ratio (RR) for total relapses; local-regional relapse; distant relapse; contralateral breast cancer; second primary malignancy, total death; death due to breast cancer; early stopping of trastuzumab due to AEs; cardiotoxicity; neutropenia; diarrhea; vomiting; and skin/nail toxicity.

b Risk of bias assessed the Jadad scale for randomized controlled trials.

c Publication bias was assessed by Egger’s and Begg’s tests.
